# Data Fusion for Driver Behaviour Analysis

**DOI:** 10.3390/s151025968

**Published:** 2015-10-14

**Authors:** Juan Carmona, Fernando García, David Martín, Arturo de la Escalera, José María Armingol

**Affiliations:** Intelligent Systems Laboratory, Universidad Carlos III de Madrid/Avda. de la Universidad 30, 28911 Leganés, Spain; E-Mails: jucarmon@ing.uc3m.es (J.C.); dmgomez@ing.uc3m.es (D.M.); escalera@ing.uc3m.es (A.E.); armingol@ing.uc3m.es (J.M.A.)

**Keywords:** human factors, CAN-BUS, driver behaviour

## Abstract

A driver behaviour analysis tool is presented. The proposal offers a novel contribution based on low-cost hardware and advanced software capabilities based on data fusion. The device takes advantage of the information provided by the in-vehicle sensors using Controller Area Network Bus (CAN-BUS), an Inertial Measurement Unit (IMU) and a GPS. By fusing this information, the system can infer the behaviour of the driver, providing aggressive behaviour detection. By means of accurate GPS-based localization, the system is able to add context information, such as digital map information, speed limits, etc. Several parameters and signals are taken into account, both in the temporal and frequency domains, to provide real time behaviour detection. The system was tested in urban, interurban and highways scenarios.

## 1. Introduction

Traffic accidents are one of the main source of injuries in twenty first century society. Most traffic accidents are caused with drivers’ inattention and misbehaviour [1]. Recent advances in computational technologies, artificial intelligence and perception technologies have led to a whole new set of applications designed to prevent these kind of accidents, by assisting the driver using so called Advance Driver Assistance Systems (ADAS) that incorporate these new technologies. ADAS applications try to detect in advance dangerous situations and warn the driver or even in certain occasions taking control of the vehicle in order to avoid the dangerous situation.

One of the key roles in avoiding dangerous situations is to identify potentially risky behaviour while driving. Modern techniques make possible the identification of these risky behaviours by means of sensors already available in the vehicle, which may include sensing devices that can easily be incorporated in everyday devices, e.g., Inertial Measurement Units (IMUs) and GPS, technologies that are available in modern smartphones, or even embedded in modern vehicles.

In this paper a novel application designed to take advantage of the on-board information available by means of a Controller Area Network Bus (CAN-BUS) sensing device, and the information retrieved from an IMU and a GPS, in order to identify potentially dangerous driver behaviours is presented. By means of data fusion techniques, all this information is combined and an estimation of the behaviour of the driver is provided. All the devices were vehicle mounted and tested in real road scenarios.

The work represents a step forward in two aspects: the first refers to the novel software sensor fusion architecture, which allows the identification of aggressive driver behaviour by means of low cost devices. The second novelty is in the hardware architecture, based on onboard information retrieved through CAN-BUS, and the specifically designed embedded sensing device information, based on IMU and GPS devices.

The rest of the paper is organized as follows: Section 2 provides an overview of the state of the art. Section 3 presents a general overview of the work. In Section 4 the hardware architecture is presented. Section 5 describes the software module. Finally results are shown in Section 6 and conclusions and future works are discussed in Section 7.

## 2. Previous Works

Driver behaviour analysis is a common topic in Intelligent Transport System and human factor studies. Several works have tried to understand the human factors involved in the driving process. Two main trends are followed for driver behaviour analysis: the first trend is related to the use of external devices, designed and mounted specifically of a vehicle for this purpose, e.g., computer vision or 3D cameras. The second trend is the usage of the available information provided by the vehicle, embedded in the available sensors, to provide information related to the state of the driver and his/her behaviour.

The first set of works achieves driver monitoring by the use of external data acquisition devices that provide further information to the system. In the work of Pelaez *et al.* [2], driver gaze is identified based on a low cost sensor (the Kinect from Microsoft), and 3D point cloud matching based on the Iterative Closest Point (ICP) method. In the work presented by Heo and Savvides [3], two cameras are used to provide frontal and profile accurate 3D face modelling and 2D pose synthesis. Murphy-Chutorian and Trivedi [4] performed 3D head pose estimation based on Localized Gradient Orientation (LGO) and Support Vector Regressors (SVRs). Oyini Mbouna *et al.* [5] provided model-based movement tracking based on optical flow. In the work presented by Garcia *et al.* [6], infrared cameras are used to identify the eye location. Li *et al.* [7] performed feature extraction from the camera used together with several biological parameters, such as percentage of eye closure, quantity of eyed closed and the Current Car Position (CCP)). Commercial eye tracking systems such as [8] and [9] are mainly based on stereo systems.

Other driver monitoring systems, such as the one described by Papadelis *et al.* [10], require measurements of biomedical signals. This requirement makes them less reliable due to the fact that these intrusive methods lead to driver behaviour changes, reducing the relevance of the measured data. Besides the lack of comfort hinders their generalization and usage in commercial applications.

The use of on-board sensors already available in the vehicle to analyze driver behaviour is a low cost and powerful alternative to the aforementioned systems. Modern vehicles include a wide variety of sensors and communication devices, which provide a large amount of data that can be used to identify specific driver behaviour, among other human factors. Some of these technologies are already available in the market, with applications such as recommended shifting points, which provides information to the driver about when to perform the gear shifting manoeuvre in order to save fuel and maximize the engine response. Other examples can be found in literature, for example, Wakita *et al.* [11] provided driver identification based on driving pattern information. Takei *et al.* [12] and Krajewski *et al.* [13] discussed driver fatigue identification based on steering wheel movement. Choi *et al.* [14] described a first attempt to perform driver behaviour analysis based on CAN-BUS information. By using Hidden Markov Models (HMM) action identification (event detection), distraction detection and driver identification, the authors reported success rates ranging from 30% to 70% according to the number of unique conditions. Al-Doori *et al.* [15] utilized a CAN-BUS information-based system and fuzzy logic in order to extend the range of electric vehicles.

The availability of modern smartphones with advanced sensing devices has led to the development of advanced applications that use these devices to provide driver analysis. For example, Johnson and Trivedi [16] provided driving style recognition by Dynamic Time Warping (DTW) and smartphone based sensor-fusion, Castignani *et al.* [17] used fuzzy logic to identify risky manoeuvres and improve driver efficiency, while Diaz Alvarez *et al.* [18] used neural networks to improve the efficiency in electric vehicles, and Eren *et al.* [19] used DTW to identify the risky behaviour of a driver through using a smartphone. Li and Busso [20] and Jain and Busso [21] fused the CAN-BUS information with other information sources, in the first case with a microphone array and video cameras, and in the second with a frontal camera. Both achieved driver behaviour analysis with different accuracy (*i.e.*, the first method provides approx. 40% positive detection, while the second provides up to 78.9% positive detection).

**Figure 1:**
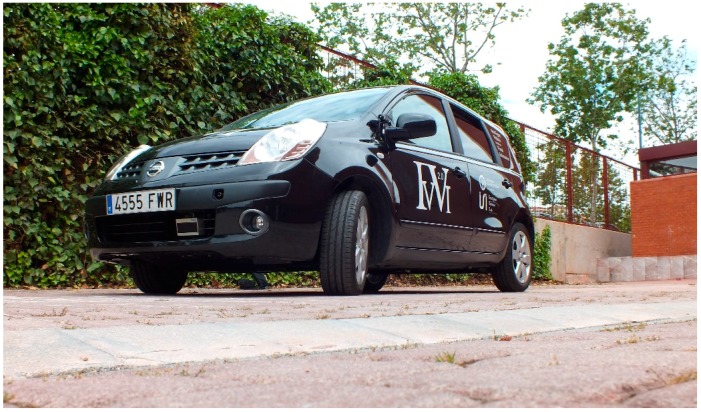
Intelligent Vehicle based on Visual Information (IVVI) 2.0 research platform.

The presented paper provides a step forward in the field of driver behaviour analysis. The data is retrieved from the available technologies in the IVVI 2.0 platform [22] (Figure 1). These technologies include the CAN-BUS monitoring system, as well as an advanced GPS system, equipped with an IMU. The latter is not typically included in commercial vehicles, however modern smartphones includes similar technologies, which combined with the CAN-BUS information would allow the proposed algorithm to be available in any vehicle.

## 3. General Overview

**Figure 2:**
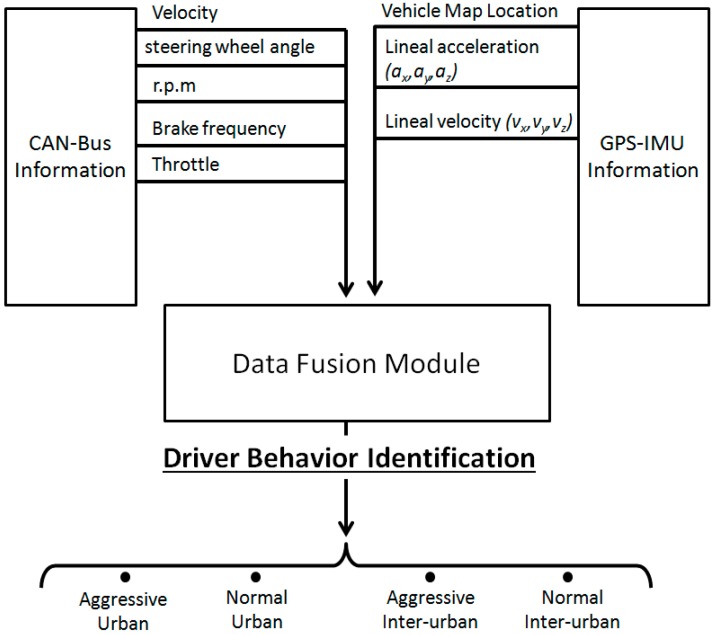
Information flow provided by CAN-BUS and GPS-IMU ground-truth device.

**Figure 3:**
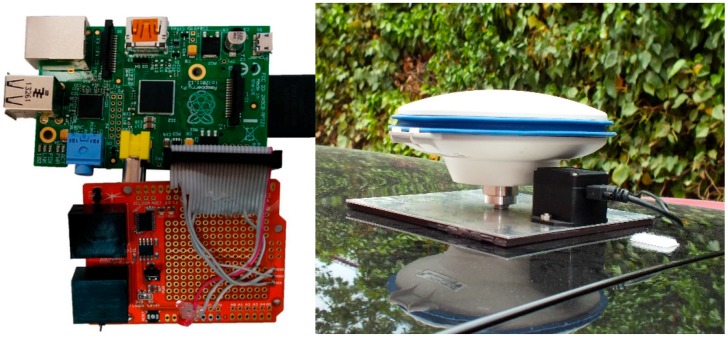
Embedded sensing devices. (**a**) Raspberry Pi and the CAN-Bus device (CANdiy-shield board from Watterott Electronic, Leinefelde-Worbis, Germany) for CAN-BUS information retrieval; (**b**) IMU and GPS installed in IVVI 2.0.

Figure 2 depicts the overall information flow, obtained from the different available sensing devices. CAN-BUS information is used to retrieve information about both driver behaviour (brake use frequency, throttle usage) and vehicle state (engine rpm, velocity, steering angle ...). All this information is retrieved via the designed embedded system, based on a Raspberry Pi device, connected to a CAN-BUS decoder. IMU information and GPS data are provided by the sensing device presented in [23] and also displayed in Figure 3b.

Figure 2 depicts all the information sources available in the system, together with the information that they are able to provide to the application. The retrieved CAN-BUS information provides full information about the vehicle state, including velocity, steering wheel angle, braking frequency and percentage of throttle pedal pressed. Further information is also available, such as the state of lights, air conditioning, *etc*., however this information was discarded as it did not represent relevant data for the application. On the other hand, the GPS-IMU module is able to provide accurate localization based on GPS with enhanced capacities and inertial information, such as acceleration and velocity. Among this information, GPS and acceleration proved to be the most useful, since velocity was already provided by the vehicle. Section 5 analyses all these sources providing full description of the signals and the information that can be inferred from each of them.

The work presented in this paper has two different parts: the hardware module and the software module. The hardware module is based on an embedded system, which is able to retrieve all the necessary information in real time. Two different modules are designed: the first is based on an embedded low-cost platform, Raspberry Pi 2, with a shield as CAN-Bus adapter. It is able to retrieve, read and write in the CAN-BUS (Figure 3a) of the vehicle (IVVI 2.0) in real time. The second unit is a GPS system with IMU (Figure 3b).

The software module is a data fusion architecture that integrates the CAN-BUS information (*i.e.*, throttle, rpm, brake pedal, velocity and steering angle) and GPS + IMU information. By means of this data, driver behaviour is identified.

**Figure 4:**
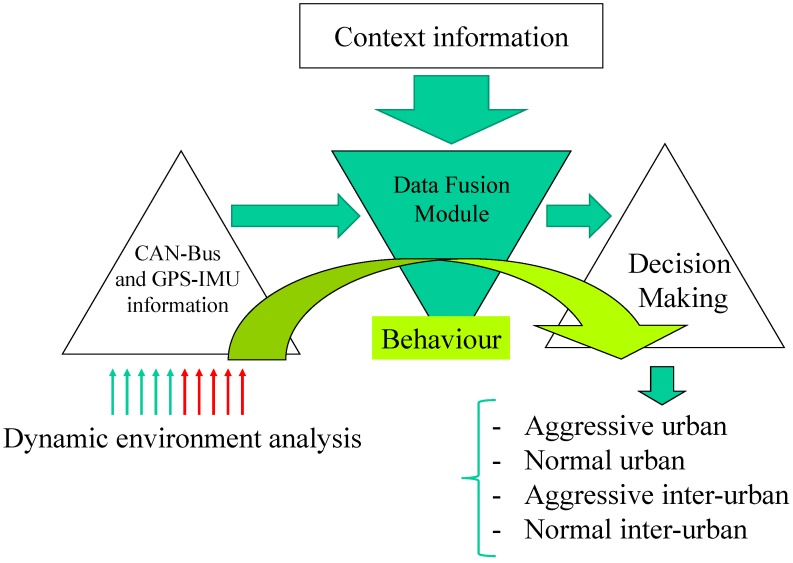
Architecture description of the expert system, information and knowledge flow to provide driver behaviour classification.

Driver behaviour is divided into aggressive and normal. Furthermore, given the specificity of the behaviour of the driver within the city, and in interurban scenarios. Four classes are defined: aggressive urban, normal urban, aggressive interurban and normal interurban. Figure 4 depicts the general software architecture that make up an expert system to obtain the driver behaviour classification. This work focuses mainly on presenting the hardware and software technology which allows driver behaviour identification. The available variables are identified, and a complete study is provided, in order to establish the values that allow the classification among the different behaviours.

Furthermore, we also present the architecture that uses a crisp ruled-based system, where the information injected into the system is explained along the manuscript, using the values that differentiate the driver behaviours. This information proves the usability of the presented technology for driver behaviour analysis. The classification is shown in the result graphs of the manuscript where the values that are embedded in the rules can be observed to establish the decision making process.

## 4. Hardware Architecture and Data Acquired

As mentioned, the hardware module is composed by two devices: the CAN-BUS communication monitor and the GPS + IMU system. Each of the systems has specific behaviour traits that are detailed as follows.

### 4.1. CAN-BUS Communication System

The designed device is based on a low cost microcomputer device, Raspberry Pi 2, and a CAN-BUS adapter. This approach allows full communication monitoring, which allows both data send and receive. The device is connected to the OBD-II port of the vehicle which allows the retrieval of the information regarding to the internal parameters of the vehicle in real time. Among the data available, Table 1 lists the data used for driver monitoring. All the information is retrieved via the high speed CAN-BUS at 500 kbps. The incorporated microcontroller permits fast and intelligent data retrieval, which allows several configurations. The software designed to run in the system provides both on-line, and off-line data processing:

Off-line data processing stores all the communication in a text file, which can be analysed later. The data format was designed to be compatible with all the main processing software.

**Figure 5:**
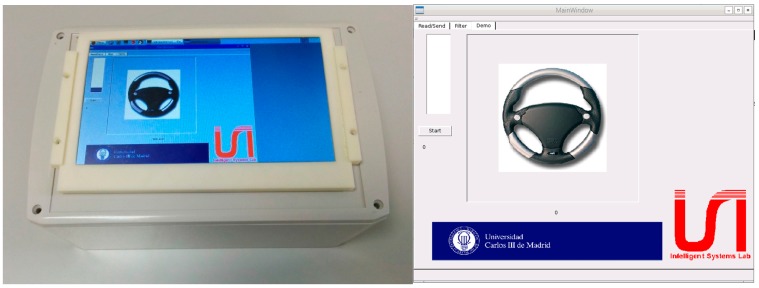
Full driver monitoring system module (**a**) system with the display mounted; (**b**) Example of real time steering wheel angle and throttle display.

On-line data processing has two options, which were designed in order to allow on-line data retrieval and display. The first option is a self-designed software that displays the CAN-BUS information in real time (Figure 5b). The second option is the real time information delivery, which is sent to the IVVI 2.0 server via Ethernet. The IVVI 2.0 Server architecture is based on the Robotic Operative System (ROS) [23], thus the information retrieved by the CAN-BUS is provided within the ROS platform.

**Table 1:** Data acquired from the CAN-BUS.

Data	Units
Vehicle lineal velocity	(km/h)
Revolutions per minute	(r.p.m.)
Brake pedal	Binary data (pedal pressed or not)
Throttle pedal	(% of pedal pressed)
Steering wheel angle	(degrees)

In order to allow the configuration of the system and the real time display of the information from the vehicle, a touch-screen was added to the system (Figure 5a). With this display, the system can be configured in real time, and monitor communications, and some vehicle parameters can be checked in real time as well (Figure 5b). Finally the system is installed in a box, which is equipped with a fan. In order to allow in-site operation, the touch screen is included in the box by the use of a 3D printed frame. The final result, shown in Figure 5b allows the manual operation and the interconnection with the vehicle architecture.

### 4.2. GPS and IMU Subsystem

This subsystem is presented in this section. The first device is a Differential Global Positioning System (DGPS), which is composed of a base station that transmits differential corrections in real-time, and a moving receiver that is integrated in-vehicle to provide the position of the vehicle in different environments; such as, urban or motorway environments, among others. The second device is an Inertial Measurement Unit (IMU), which has embedded accelerometers and gyroscopes.

The two receivers, base station and in-vehicle device, are two NovAtel OEMV-1G, which offer GPS + GLONASS L1 tracking and provide positioning even in complex environments such as urban canyons. These devices have the property of being embedded in a compact enclosure (FlexPak-G2-V1G) for outdoor applications. The main characteristics of the receivers are the following: (i) Pulse Aperture Correlator (PAC) with multipath mitigation which offers multipath-resistant processing at high data update rates, and (ii) high acquisition and re-acquisition times that allow it to operate in urban environments, where frequent signal interruptions can be expected. Moreover, the advantage of the NovAtel antenna (GPS-701-GG), from NovAtel (Calgary, Canada), used in base and on-vehicle, is multipath rejection.

The in-vehicle receiver calculates positioning based on two performance modes. The first mode is the basic positioning solution, called single point position mode (SINGLE mode), where all available GPS satellites are used in the position solution without differential corrections. The second mode is differential mode (DGPS), where the base station is positioned in an accurately known location that transmits the range corrections to the in-vehicle receiver. In this work, the configuration of the update rate associated with SINGLE or DGPS modes has been selected 5 Hz, where the in-vehicle receiver automatically switches between both modes, DGPS mode has priority if appropriate corrections are correctly received. Moreover, this system has been selected to use L1 C/A-code data (pseudoranges) for differential solution due to its advantages in urban, inter-urban and motorway environments instead of using carrier-phase DGPS. The first disadvantage of the carrier-phase DGPS, such as Real-Time Kinematic (RTK), is the age of RTK data, where a delay from 5 to 60 s is desirable, whereas the restriction for pseudorange differential age is a broad delay from 2 to 300 s. The second carrier-phase disadvantage is the initialization process, which is necessary under optimal conditions, and cm-level precision is reached after 30 to 40 min. The third disadvantage is that if the receiver uses less than four satellites in RTK mode after the initialization process, the receiver must restart this process to reach again cm-level precision. The fourth disadvantage is the line between base and in-vehicle receiver (baseline) for good accuracy in RTK mode, which is desirable to be less than 15 km. The single-frequency Global Navigation Satellite System (GNSS) receiver, model: OEMV-1G (from Novatel, Calgary, AB, Canada) used in this work, can reach RTK 20 cm position accuracy after static convergence, and RTK 2 cm after convergence and maximum baseline of 3 km. However this DGPS system, using L1 C/A-code data, requires only a single epoch of common data, which is an advantage in urban, inter-urban and motorway environments, where the recovery time of the DGPS accuracy is minimized. Then, carrier-phase DGPS is relegated to high-accuracy applications in ideal conditions, and the experiments of this work are performed with a DGPS mode using L1 C/A-code data for differential solution, where accuracy is less than 1 m.

The second device is an IMU, a 3DM-GX2, from MicroStrain (Williston, VT, USA) which integrates a triaxial accelerometer, triaxial gyroscope and triaxial magnetometer. The IMU data are highly appreciated in this work to compare it with data from CAN-BUS, that is, it allows the establishment of the IMU ground-truth data to be compared with CAN-BUS data. The IMU data, accelerometers and gyroscopes measurements, are acquired at 100 Hz.

## 5. Driver Behaviour Analysis Software Module

Signals are merged and fused together based on an intelligent expert approach. The intelligent approach is based on the use of signal descriptors, which identifies specific patterns in the driver’s behaviour. These specific patterns are related with the signals shown in Table 1. The descriptors are obtained in both time and frequency domains, which are used later to train the system for the intelligent detection system.

### 5.1. Accurate Localization and Digital Maps

**Figure 6:**
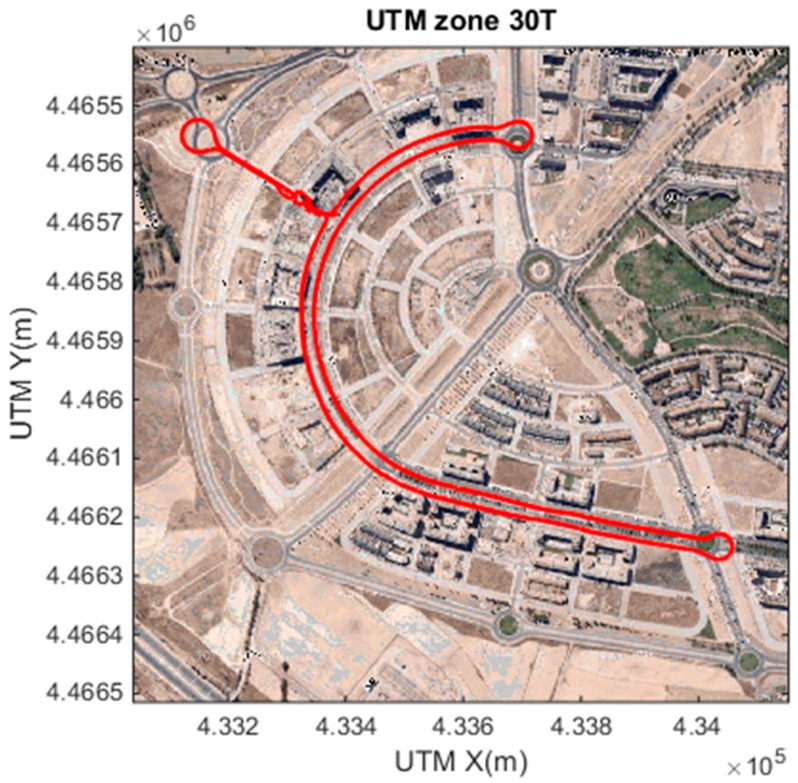
Digital map localization example, used to configure the system based on context information.

The accurate localization, based on the GPS + IMU, presented in [2] is used to provide reliable urban localization. By means of digital maps and this accurate localization, the system identifies when the vehicle is driving in urban environments, and adapts the configuration of the system to the situation. Figure 6 shows the vehicle trajectory at the urban environment where the experiments have been performed.

### 5.2. Time Domain Descriptors

Time information related to the statistical value of the signal is used, and information such as the mean value, or the peak value was used to identify the behaviour patterns. This information was integrated into a given time window, configurable by software that gives an estimation of the driver behaviour in the defined window.

Two values were provided by the fusion system; instant behaviour and aggregate behaviour. The first is based on the information provided in real time, and checks the proper behaviour of the driver given specific parameters. This information takes into consideration context information and is based on an expert system. Events are displayed as acoustic and visual alarms to the driver indicating the improper behaviour, e.g., strong longitudinal acceleration from a static position triggers an alarm that indicates aggressive behaviour.

Aggregated information provides driver estimation of the behaviour according to statistical values in a given time. This information is not based in the sole information of a single signal, but on the combination of the different signals, described in descriptor vectors based on both the time and frequency domain. All the information is combined in the final stage. Statistical and time information is different, according to the signal available, as presented in Section 5.2.1 and Section 5.2.2.

#### 5.2.1. CAN-BUS Based Descriptors

(1) Vehicle Lineal Velocity

The signal represented as *v*[*t*], and expressed in kilometres per hour [km/h]. This information is essential to identify important behaviours such as (maximum) speed limit infringements. Aggregated information such as the mean can be used to identify the speed over a specific period of time and standard deviation can be used to identify high changes of velocity, which may correspond to erratic and aggressive behaviour:

Max amplitude [km/h]:

*v*[*t*] _max_ = max(*v*[*t*]) (1)

Mean value [km/h]:

(2) $$\bar{v}=\frac{1}{N}\overset{N}{\underset{1}{\sum }}v\left[t\right]$$

Standard deviation [km/h]:

(3) $$\sigma_{v}=\sqrt{\frac{1}{N}\overset{N}{\underset{1}{\sum }}{\left(\bar{v}-v\left[t\right]\right)}^{2}}$$

Median value [km/h]:

(4) $${\tilde{v}}_{\text{even}}\;={\left(\frac{N}{2}+1\right)}^{th}term$$

(2) Revolutions per Minute

Signal represented as *r*[*t*], and expressed in number of revolutions per minute [r.p.m.]. This information is valuable to identify specific behaviour. In this application, it was used to identify aggressive behaviour through the identification of extreme use of the vehicle engine (*i.e.*, high values). However, this information, together with the gear use information, can be used to identify other important parameters, such as fuel consumption. Here, the maximum value can be used to identify instantaneous misbehaviour, and aggregated values can be used to differentiate a continuous misuse (where our method uses the mean and the median). Furthermore, standard deviation can be used to detect high change rate of the revolutions, which identifies fast and erratic movements, and in many occasions, implies aggressive behaviour. Here, formulation is similar to Equations (1)–(4), thus no further formulation is needed:

Max amplitude [r.p.m.]:

*r*[*t*]_max_ (5)

Mean value [r.p.m.]:

(6) $$\bar{r}$$

Standard deviation [r.p.m.]:

(7) $$\sigma_{r}$$

Median value [r.p.m.]:

(8) $${\tilde{r}}_{\text{even}}$$

(3) Brake Pedal

It is a signal represented by *b*[*t*] which is a binary signal with a value of 0 when the pedal is not pressed and 1 when it is pressed. Strong braking actions can identify aggressive manoeuvres and are a danger to road safety. Besides, the successive repeated use of the pedal is a sign of erratic driving. All this information can be inferred from the study of the braking manoeuvres, however the information available was limited to pedal pressed or not, which is not enough to identify strong braking manoeuvres. Further information, such as the one provided by the IMU, is combined with the brake pedal information, in order to provide identification of these strong braking manoeuvres:

Braking time (time pedal pressed) [%]

(9) $$bt=\frac{time\;pedal\;pressed}{total\;time}$$

Braking frequency (times pedal pressed) [Hz]

(10) $$bf=\frac{\#\;times\;pedal\;pressed}{total\;time}$$

(4) Throttle Pedal

The percentage of the throttle pedal pressed, represented as *thr*[*t*] and expressed in a percentage [%]. The throttle pedal press percentage provides direct information of the driver intention, as a high percentage of pedal pressing identifies a clear intention to exceed the limits provided by the vehicle, so here maximum, mean and median can provide important information. Besides, standard deviation identifies erratic behaviours:

Max amplitude [%]:

*thr*[*t*]_max_ (11)

Mean value [%]:

(12) $$\bar{\text{thr}}$$

Standard deviation [%]:

(13) $$\sigma_{\text{thr}}$$

Median value [%]:

(14) $${\tilde{thr}}_{\text{even}}$$

Acceleration frequency [Hz]:

(15) $$tf=\frac{\#\;times\;pedal\;pressed}{total\;time}$$

(5) Steering Wheel Angle Movement

Signal with the angular velocity of the steering wheel, represented as *ω*[*t*] and expressed in degrees per second (°/s). The steering wheel information may not provide information by itself, since the absolute degree of the movement usually remains constant whether the driver behaves in an aggressive way or not while driving along the same roads. However, steering wheel velocity may provide significant information. Fast changes of lanes, strong lateral movements, may be identified by fast steering wheel movement. Here, the study of all different values, such as maximum, mean, median and standard deviation, together with the lateral acceleration explained later, are important to identify such movements:

Max amplitude (°/s):

*ω*[*t*]_max_ (16)

Mean value (°/s):

(17) $$\bar{\omega }$$

Standard deviation (°/s):

(18) $$\sigma_{\text{ω}}$$

Median value (°/s):

(19) $${\tilde{\omega }}_{\text{even}}$$

#### 5.2.2. IMU Based Descriptors

##### Linear Acceleration

Acceleration represented as *a*[*t*], and expressed in meters per square seconds (m/s^2^). Three signals are available, a_x_, a_y_, a_z_, all of them corresponding to a different axis. All of them have similar descriptors presented in the following equations. Accelerations are important to measure the comfort level of the vehicle occupants. Besides, combined with some of the aforementioned information, such as steering wheel movement, or brake pedal information, it is possible to identify the behaviour of the driver. Here, the two most important were lateral (x) and longitudinal (y). Although vertical acceleration was included in the information retrieved, it did not provide extra information, and thus it was not used for identification:

Max amplitude (m/s^2^):

*a*[*t*]_max_ (20)

Mean value (m/s^2^):

(21) $$\bar{\text{a}}$$

Standard deviation (m/s^2^):

(22) $$\sigma_{\text{a}}$$

Median value (m/s^2^):

(23) $${\tilde{\text{a}}}_{\text{even}}$$

### 5.3. Frequency Domain Descriptors

**Figure 7:**
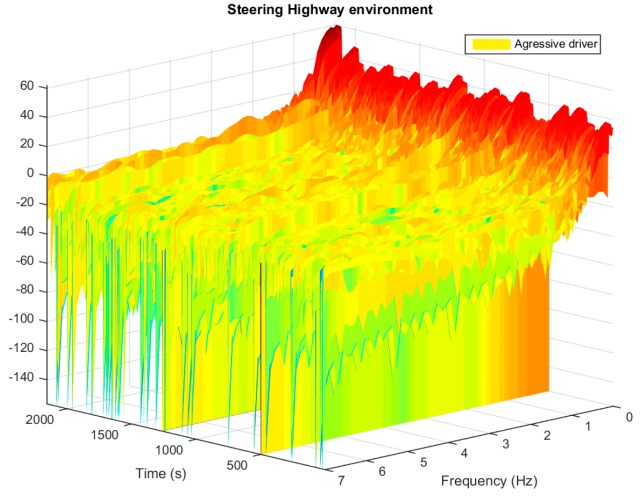
Spectral analysis of the steering wheel movement in highway environment. Signal power (db) is represented in the vertical axis, frequency (Hz) and time representing the moment where the spectral analysis is calculated, are represented in horizontal axis.

**Figure 8:**
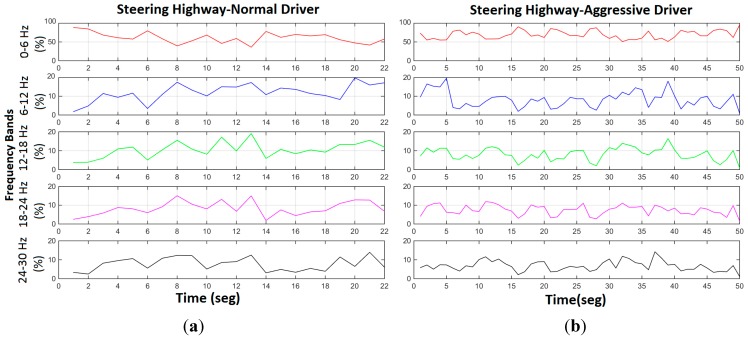
Frequency interval evolution of the steering wheel movement in a highway environment: (**a**) normal driver, (**b**) aggressive driver. The vertical axis represents the percentage of power on this part of the spectrum.

In order to provide spectral information, frequency information is included as a descriptor, based on the frequency information of the signal. The frequency descriptor is the basis in the spectral analysis of the signal within the corresponding window. This spectral analysis is shown in Figure 7, based on a specific time window. Here the third dimension depicts the evolution along time of this spectral analysis. Relevant frequency information provides useful information for driver behaviour analysis, such as working frequency identification, and fast movements. However, this information is too extensive to be processed. Thus information obtained with the spectral analysis has to be processed by means of frequency descriptors. These frequency descriptors summarize the relevant information provided by this spectral analysis. This way, the information obtained and represented in Figure 7, which is difficult to process, can be converted into Figure 8 by these frequency descriptors. Once the spectral analysis is performed, the frequency domain is divided into five sections. Each section corresponds to a continuous frequency interval. Once the spectral signal is divided into five different intervals, the percentage of the power of the signal in each interval is calculated, returning five different descriptors. As these descriptors are calculated based on a time window, which is overlapped, and these descriptors evolve in time, as depicted in Figure 8. Therefore, Figure 8 represents a bidimensional representation of Figure 7, based on the aforementioned spectral descriptors which summarize the spectral information contained in the signal.

### 5.4. Context Information

In addition to the aforementioned signal information, context information is used in this approach. Context information refers to on-line or off-line information, which can be retrieved from the context situation and helps to enhance the estimation. In this case, the context information is exploited to understand the real situation of the vehicle, consequently providing comparison of the vehicle situation in relation to the ideal. Among the context information, the following points should be addressed.

#### 5.4.1. Maximum Recommended Acceleration

For both braking and accelerating manoeuvres, the maximum acceleration is identified according to human factors research. This way, the real time values obtained from the IMU to identify aggressive instant behaviour are used to identify uncomfortable movements. According to [24], stable non-emergency accelerations in the range 0.11 g to 0.15 g fall in the “acceptable range” for most studies in acceleration comfort of ground vehicles. It is unlikely that values of jerk larger than 0.30 g can be acceptable as comfortable. This way, movements higher than 0.3 g were identified as non-conformable, and 0.5 g ones were identified as aggressive instantaneous behaviours. This values were both identified in order to allow a detection of aggressiveness in two ways. First, an uncomfortable movement repeated in time would lead to the identification of the behaviour as aggressive, the second allows an instantaneous identification by the detection of an aggressive manoeuvre. $a_{y}>3\;\left[\frac{m}{s^{2}}\right]$, non-conformable movement, $a_{y}>5\;\left[\frac{m}{s^{2}}\right]$, aggressive movement.

#### 5.4.2. Maximum Allowed Speed

Based on the digital map information and the accurate localization, the vehicle provides information of the maximum speed allowed on the road. This way it can be compared with the measured value. This value is also used as descriptor in the algorithm according to Equation (24):

(24) $$\bar{dv}=\frac{1}{N}\overset{N}{\underset{1}{\sum }}\left(v_{r}-v\left[t\right]\right)$$

where *v_r_* represents the maximum velocity allowed in the road. Furthermore an index (*i*_vtp_) is provided indicating how many times the maximum velocity is trespassed during a given period.

#### 5.4.3. Urban/Interurban Location

GPS localization is used to indicate whether the vehicle is located within an urban environment or not.

## 6. Tests and Results

With the information presented in Section 5, an expert intelligent system is developed. This system provides both event detection and aggregate identification. Both detection systems are trained to identify specific patterns provided by the aforementioned descriptors.

### 6.1. Configuration Tests

The performed tests involved three different scenarios: downtown urban environment (dense traffic), suburban scenario (clear traffic) and highway scenario. In every scenario different behaviours are trained, both normal and aggressive. The collected data was used to identify the patterns of aggressive manoeuvres, and normal manoeuvres over the three scenarios. All possible manoeuvres were tested, identifying the specific values which describe every situation. This information was used to create the crisp ruled-based system which identifies the behaviour patterns of aggressive drivers in the three scenarios.

**Figure 9:**
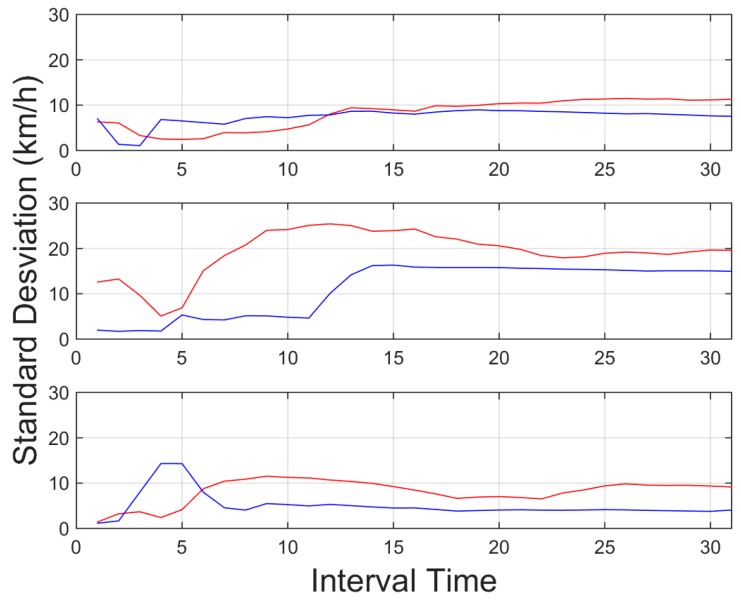
Standard deviation for vehicle velocity, for an interval time = 20 s. Red indicates aggressive driver, and blue line, a normal driver. Top downtown environment, centre interurban environment and bottom highway environment.

Figure 9 depicts the standard deviation for vehicle velocity, the obtained values show higher values in the standard deviation for the vehicle velocity in aggressive driver behaviour. Here the environment is important since the value is higher in interurban environments with higher velocities, than downtown urban environments where the velocities are lower, thus the obtained values are lower. Here different threshold were used for each environment. The environment identification is obtained based on GPS and digital maps. Besides as it can be observed, this information is more helpful in highways and interurban environments where the ranges of velocities available are wider, than in urban environments where these velocities are more limited.

**Figure 10:**
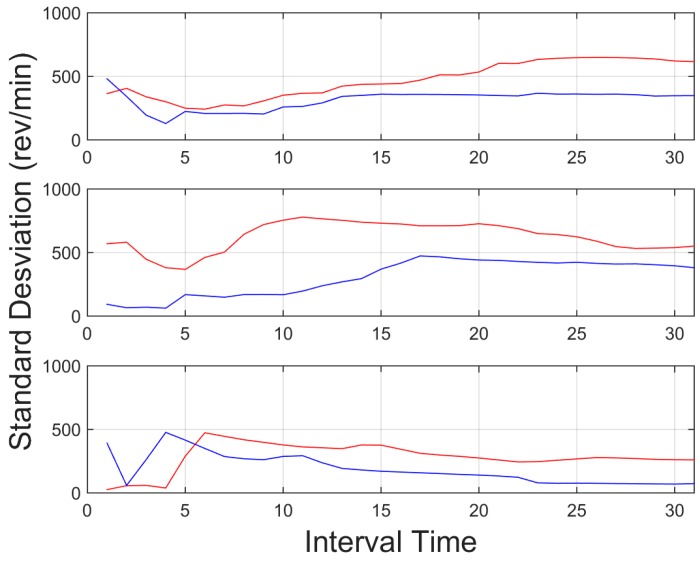
Standard deviation for revolutions per minute (r.p.m.) of the engine, for an interval time = 20 s. Red indicates an aggressive driver, and blue line, a normal driver. Top downtown environment, centre interurban environment and bottom highway environment.

Figure 10, on the other hand, shows the standard deviation of the vehicle engine r.p.m. These data allowed the identification of the behaviour, since the aggressive behaviour showed a great variation in the use of the engine, with high changes in the revolutions and thus representing a higher standard deviation.

Urban environments with more stop and go manoeuvres derives in higher changes in the engine r.p.m., thus standard deviation proved to be a good tool on this environment, as well as interurban environment. On other scenarios, where the stops are less frequent, such as highways environments, absolute values, such as mean or peaks provide more information about the driver behaviour.

**Figure 11:**
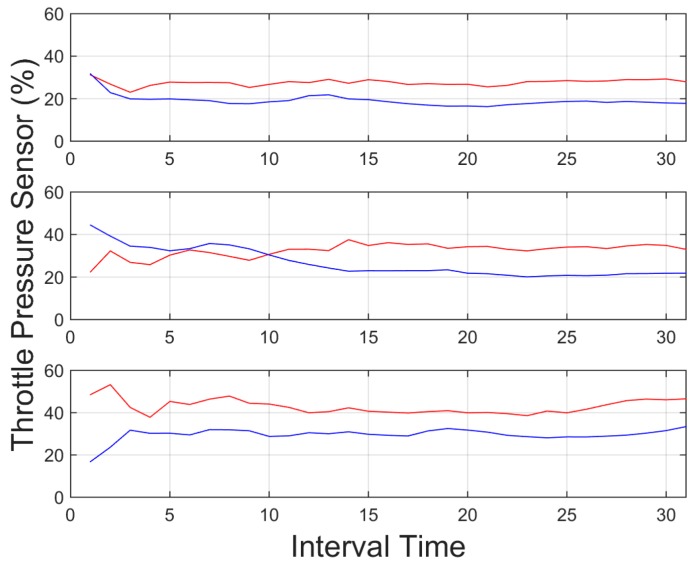
Mean percentage of throttle pressed for an interval time = 20 s. Red indicates an aggressive driver, and blue a normal driver. Top: downtown environment, centre: interurban environment and bottom: highway environment.

Figure 11 provides the mean percentage of throttle pressed during a time interval of 20 s and its evolution along time. As shown, the provided data shows a considerable higher value with the aggressive driver, almost 100% of the time whereas for normal driving conditions, the driver showed a considerably lower value. This difference is higher in urban environments than in highway environments, as shown in the figure, which is due to the fact that in highway environments, the driving process is more stable, thus the percentage of pedal pressed is more continuous.

**Figure 12:**
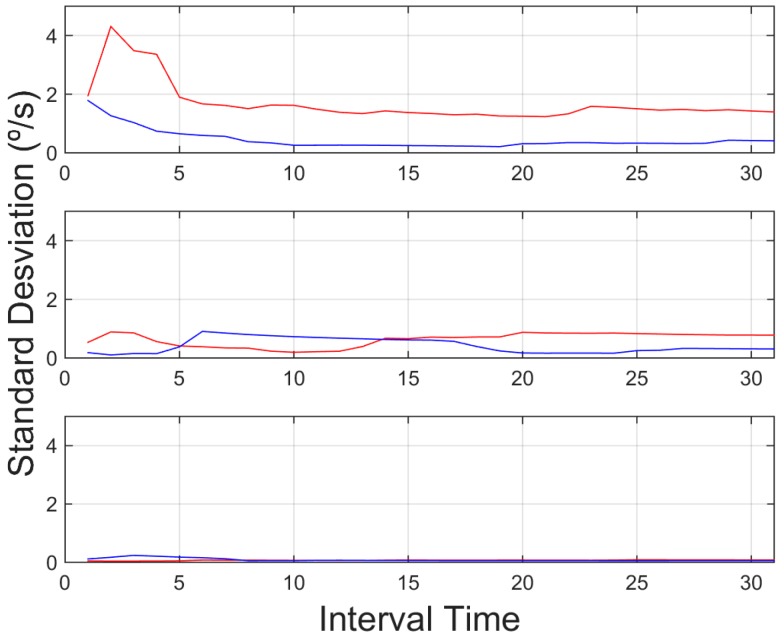
Standard deviation of steering for an interval time = 20 s. Red indicates an aggressive driver, and blue a normal driver. Top: downtown environment, centre: interurban environment and bottom: highway environment.

**Figure 13:**
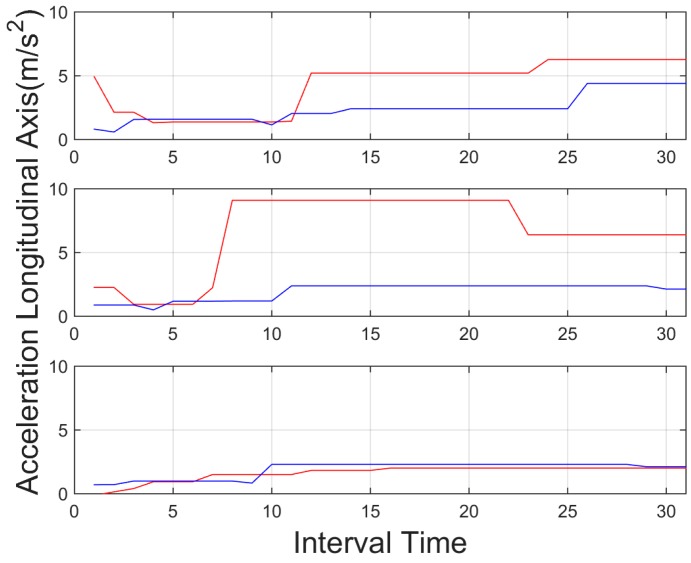
Local peaks for acceleration of longitudinal axis, for an interval time = 20 s. Red indicates an aggressive driver, and blue a normal driver. Top: downtown environment, centre: interurban environment and bottom: highway environment.

**Figure 14:**
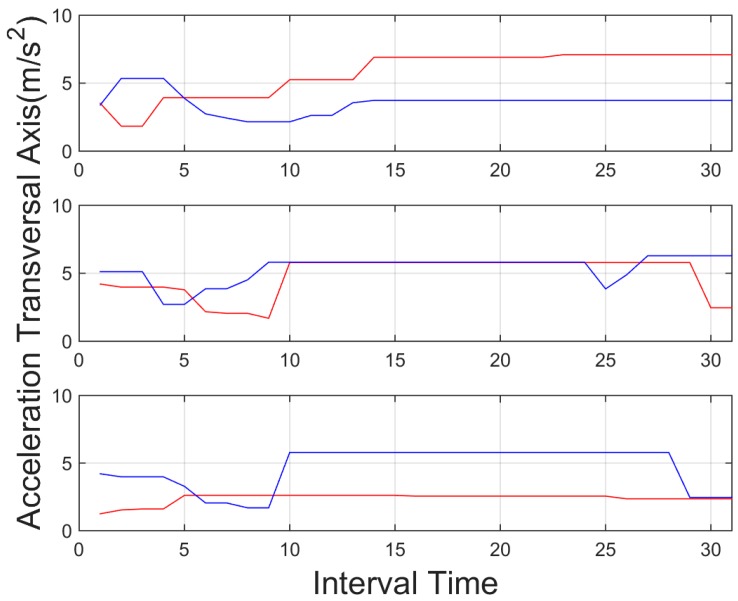
Local peaks for acceleration of transversal axis for an interval time = 20 s. Red indicates an aggressive driver, and blue a normal driver. Top: downtown environment, centre: interurban environment and bottom: highway environment.

Further information is also useful for aggressive driver identification. In Figure 12, the standard deviation of the steering wheel movement (angular velocity), shows a higher value again for the aggressive behavior in all environments, even in highway environments, with lower lateral movements, proving the significance of identifying these kinds of behavior in most of the cases. Accelerations, on the other hand, didn't show stable values that could be used in the aggregated information. Tests showed that the identification of peak values in the aggregated time is not enough information to identify these behaviors. As depicted in Figure 13 and Figure 14, these values showed a significant oscillation that is not enough to use them in the aggregated time. On the other hand these values can be used to identify instant behaviour identification. By identifying strong lateral or longitudinal accelerations, the system identifies the corresponding manoeuvres as aggressive. In order to allow the system to include this kind of information, it counted the number of times these signals are triggered. Therefore, it can identify over time the driver who showed this behaviour as aggressive.

### 6.2. Field Tests

**Figure 15:**
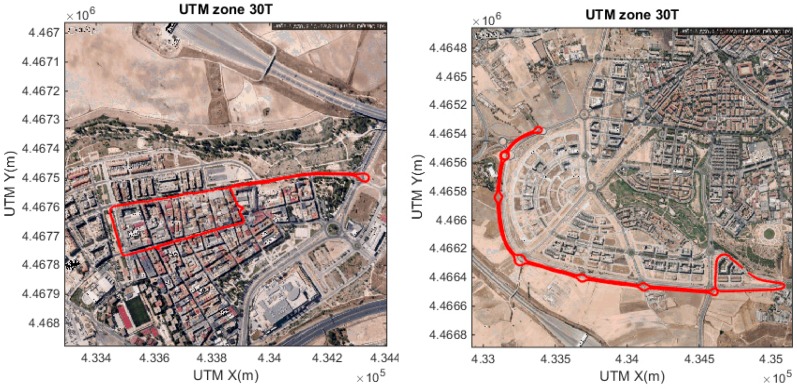
Driving scenarios used for the test, urban (**left**) and interurban (**right**).

A total of 20 further test were performed with 10 different subjects in order to test the algorithm. Then, the different thresholds and feature identified in the previous sections were used to identify the aggressive or non-aggressive behavior, in both urban and interurban scenarios. These tests involved an urban scenario and interurban scenarios (Figure 15). The test involved one aggressive and one normal behavior per subject. Although the drivers were asked to drive aggressively all the tests were done obeying all the traffic regulations.

Each scenario involved around 10 minutes, and the classification was based on the 23 descriptors shown in Section 5.2 and different test were performed. The configuration tests explained in Section 6.1 helped to identify the rules and the thresholds to define an aggressive behavior. When a given number of descriptors provides aggressive behavior identification, the system identifies the behavior as aggressive. In all the tests performed the system was able to identify the aggressive, and non-aggressive driving behavior after around 1 min of driving. As stated before, the beginning of the sequence did not provide enough information of the driving behavior, thus no accurate identification was provided in this part of the sequence.

**Figure 16:**
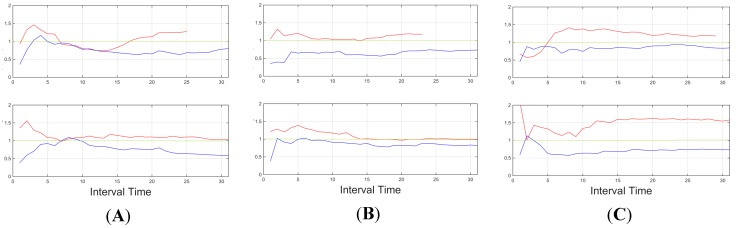
Lateral acceleration (standard deviation), for three subjects (**A**, **B** and **C**). In interurban scenarios (down) and urban scenarios (up). The threshold for this feature is identified in green.

**Figure 17:**
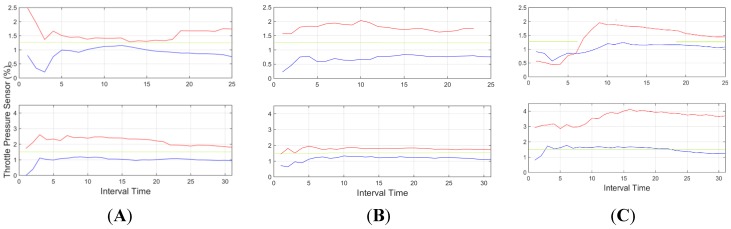
Throttle (standard deviation) for three subjects (**A**, **B** and **C**). In interurban scenarios (down) and urban scenarios (up). Threshold for this feature is identified in green.

**Figure 18:**
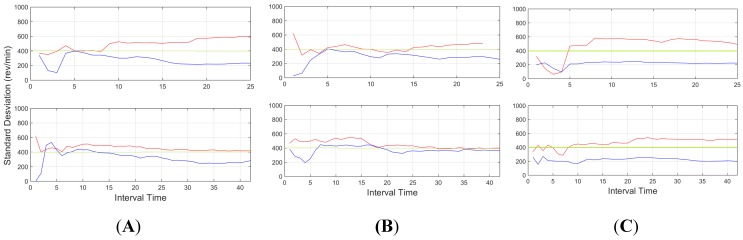
R.p.m. (standard deviation), for three subjects (**A**, **B** and **C**). Here, all the drivers reach a level over 400 r.p.m. in the vehicle while performing aggressive driving.

Figure 16, Figure 17 and Figure 18 show some of the signals obtained during these test, focusing on three subjects. The three subjects were selected as a representative set of the whole experiment. The discussion of the processes followed to identify the subject are also provided.

In Figure 16, lateral acceleration (standard deviation) is displayed. Subjects B and C provide enough information to identify aggressive behaviour, however, for subject A, this information is not enough in an urban environment, but it is identified.

In Figure 17, throttle information (standard deviation) is displayed for the three same subjects. Here subjects A and B are identified as being over the selected threshold, however, for subject C normal behaviour falls over the threshold in several occasions during interurban mode. This more aggressive normal driving during acceleration was inconclusive for conclusive aggressive behaviour identification since all other features did not identify it as aggressive (see Figure 16 and Figure 18).

**Figure 19:**
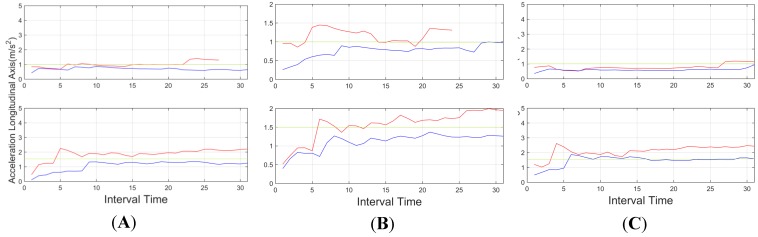
Longitudinal acceleration (standard deviation), for three subjects (**A**, **B** and **C**). Here, all the drivers reach to a level over 400 r.p.m. in the vehicle while performing aggressive driving.

Figure 18 shows the revolutions per minute information (standard deviation). Here all three subjects were above the threshold, and only subject B presented some periods of time where it fell under the threshold, but in these situations, the other features (see Figure 17 and Figure 19) where enough to provide an accurate estimation.

Figure 19 shows the information observed through the standard deviation of the longitudinal acceleration, again, the behaviour is clearly identified in most of the cases. Only in subject C there are some problems in the urban environment. However, in all the previous data (Figure 16, Figure 17 and Figure 18) it is possible to identify this specific scenario.

## 7. Conclusions

A novel system for driver behaviour identification is presented. The system takes advantage of the on-board information provided by the vehicle *i.e.*, CAN-BUS, and the information provided by an embedded IMU-GPS system. The application is able to identify and classify normal and aggressive manoeuvres in real time and aggregate behaviour based on a time window. The presented system provides an advanced driver behaviour monitoring system, based on the on-board information and advanced embedded sensors.

The developed system proved to be a robust behaviour identification algorithm with advanced capabilities. Thanks to the use of on-board information based on CAN-BUS, the system can be installed in any vehicle. Furthermore, the GPS and IMU sensors are embedded in many of the modern smartphones. Therefore, the presented algorithm can be used in any vehicle, providing a low-cost application, which is able to provide a full understanding of driver attitude and behaviour, detecting misbehaviour in real time.

The presented work is a step forward in driver monitoring system design by providing a robust multi-platform device based on both affordable hardware and advanced software capabilities. Furthermore the system provides a solution for driver monitoring applications, which can provide feedback for both public and private sectors about the real behaviour of a driver.

As proved in the Results section, the application represents a novel contribution in both the software and the hardware architecture. The software architecture presented is based on data fusion techniques and temporal and frequency descriptors, merged in a crisp ruled-based expert system. On the other hand, the hardware device is the second contribution of the paper. Thanks to the design of a novel processing unit, based on the on-board information retrieved through the CAN-BUS, and the IMU + GPS information, this system can be applied to a wide variety of current vehicles with a limited cost.

In light of the results obtained, one of the weaknesses of the presented results is the initial data comparison delay, that is, a certain time interval is required to retrieve enough data to provide an aggregate detection. As it can be observed in Figure 9, Figure 10, Figure 11, Figure 12, Figure 13 and Figure 14, during the initial period, the data can be misinterpreted due to the necessity of further analysis. However, as can be observed, over time, the data becomes stable, thus reliable behaviour identification is possible. Another important point to consider is the necessity for multiple signals in order to provide an accurate identification. Analysis based on a single signal can lead to misinterpretation, e.g., an urban driver behaving normally while driving along a closed curve; in this case strong lateral movement may be expected, however, the absence of other indicators of aggressive driving, leads to the interpretation of this movement as normal behaviour. Here, the fusion of several descriptors is very important to allow accurate behaviour identification. On the other hand, event detection, based on a single signal, may identify dangerous manoeuvres or behaviours in real time, e.g., strong longitudinal acceleration identifies strong braking actions, identifying possible dangers in the driving process in real time.

This application has great potential and direct application in several fields and markets, including insurance companies, public entities, human factor research and more. Future works will focus on the testing and development of further novel expert system-based technologies to enhance the presented work by means of advanced data fusion techniques. Besides, further capabilities will be added to the system, based on an advanced perception system developed in our laboratory, including among other features a lane departure system, and driver gaze detection, that all are already available in the IVVI2.0 research platform.
